# Manifestations of and factors influencing posttraumatic growth among Chinese Crohn’s disease patients: a qualitative exploration

**DOI:** 10.1080/17482631.2024.2422137

**Published:** 2024-11-05

**Authors:** Hong Li, Dandan Chen, Chen Zhang, Yunxian Zhou

**Affiliations:** School of Nursing, Zhejiang Chinese Medical University, Hangzhou, Zhejiang, China

**Keywords:** Crohn’s disease, posttraumatic growth, qualitative research, positive psychology, semi-structured interview

## Abstract

**Objective:**

This qualitative study aims to explore the manifestations of posttraumatic growth among Chinese patients with Crohn’s disease from a cultural perspective and to identify the factors influencing it.

**Methods:**

A descriptive qualitative research method was used. Semistructured interviews were conducted with 19 patients with Crohn’s disease from several hospitals in Hangzhou, Zhejiang Province, China, and the China Crohn’s and Colitis Foundation.

**Results:**

There were five key manifestations of posttraumatic growth experienced by Chinese patients with Crohn’s disease: improving interpersonal relationships, enhancing personal strengths, changing life priorities, expanding possibilities and gaining religious faith. Posttraumatic growth among patients was predominantly influenced by individual factors, the disease condition and social support.

**Conclusion:**

The findings regarding the manifestations of and factors influencing posttraumatic growth in Chinese patients with Crohn’s disease are consistent with those in previous studies in patients with chronic diseases. However, our study underscores the multifaceted impact of Chinese cultural characteristics on posttraumatic growth among Crohn’s disease patients. These findings can offer valuable guidance for future complex interventions and relevant clinical studies conducted within the Chinese population.

## Introduction

Crohn’s disease (CD), a form of inflammatory bowel disease (IBD), is a chronic inflammatory disorder with a complex aetiology (Flynn & Eisenstein, 2019). Its clinical manifestations primarily involve gastrointestinal symptoms, such as abdominal pain, diarrhoea, and rectal bleeding (Flynn & Eisenstein, 2019). CD is also associated with extraintestinal manifestations, including nodular erythema and ocular complications, as well as complications such as intestinal obstruction, perforation, and malignancy (Wang et al., 2010; Zeng et al., 2013). The disease course of CD is characterized by recurrent flare-ups, and there is currently no known cure (Dolinger et al., 2021). This condition imposes significant economic and psychological burdens on patients, which often leads to anxiety, depression, and/or posttraumatic stress disorder (Bernklev et al., 2006; Bisgaard et al., 2022; Taft et al., 2019). However, with a shift in psychological research has revealed that individuals with chronic illnesses can experience positive psychological changes during the disease process, leading to posttraumatic growth (PTG).

PTG refers to the positive psychological transformations experienced by individuals after struggling with highly challenging life crises (Tedeschi & Calhoun, 2004). Initially performed with acute trauma populations, PTG research has been expanded to include patients with chronic illnesses such as AIDS, cancer, and leukaemia over the past decade (Pierobon et al., 2011).

In fact, as a chronic disease, CD has been the subject of PTG-related research. Canadian researchers have conducted qualitative research to explore positive psychological changes in patients diagnosed with IBD and reported that 70% of IBD patients experienced positive psychological transformations during their illness (Skrastins & Fletcher, 2018). In China, researchers have investigated the growth experiences of young and middle-aged IBD patients and identified five themes: spiritual change, internalized supportiveness, cognitive reshaping, externalized behaviours, and future-oriented thinking (Wu et al., 2022). A longitudinal study conducted in the UK involving 152 IBD patients revealed that patients with high levels of coping efficacy, disease acceptance, and social support presented high levels of PTG (Sirois & Hirsch., 2017). Furthermore, other studies have indicated that the PTG of IBD patients is associated with disease status, patients’ perceptions of the disease, and the degree of illness acceptance (Hamama-Raz et al., 2021; Sirois & Hirsch, 2017).

While some attention has been given to the manifestation and factors influencing PTG in IBD patients, targeted research specifically involving CD patients remains scarce. Compared with patients with ulcerative colitis (UC), CD patients exhibit more complex clinical manifestations, a higher prevalence of complications, and a relatively worse prognosis (Flynn & Eisenstein, 2019; Zeng et al., 2013). The manifestations of and factors influencing PTG in this specific group may have unique characteristics.

Moreover, the aforementioned studies focused primarily on patients from Western countries, but China’s social culture and medical system differ significantly. Western societies emphasize individualism, whereas Eastern cultures, including those in China, emphasize collectivism, which affects individuals’ psychological responses to diseases (Kashyap & Hussain, 2018). In terms of the medical system, China has both public and private hospitals, with the former predominating, and healthcare is defined by traditional Chinese medicine and Western medicine paradigms (Hesketh & Zhu, 1997; Lan & Pan, 2020). A three-tier healthcare service system is utilized, which differs from Western models because of the lack of general practitioners conducting initial disease screenings and a mandatory referral system (Lan & Pan, 2020). Patients have the freedom to choose healthcare facilities at different levels (Zhang et al., 2020). Additionally, the government offers universal basic medical insurance, although insurance policies vary in terms of benefits and deductibles between provinces and between urban and rural areas (Lan & Pan, 2020). These differences may lead patients to develop varied psychological responses during treatment. Thus, it is vital to understand how PTG manifests among CD patients in China and which factors influence PTG outcomes.

Therefore, this study was performed to investigate the manifestations of and factors influencing PTG in Chinese CD patients with the aims of enhancing our understanding of psychological adaptation in this patient population and providing insights into clinical psychological interventions.

## Methods

### Study design

This study is based on a postpositivist research paradigm (Devers, 1999). The ontological assumption is that reality is “real” but can be understood only incompletely and probabilistically (Willis, 2007). An objectivist epistemological perspective was adopted, asserting that research findings are likely to approximate the truth (Willis, 2007). On the basis of these principles, a descriptive qualitative research approach was adopted, which uses everyday language to directly describe the experiences of participants or to present an event without prior theoretical commitments (Lincoln et al., 1985). It posits that realities need to be understood within specific contexts and timeframes (Lincoln et al., 1985). The aim of this qualitative approach is less theory-driven but rather seeks to offer a comprehensive summary or straightforward description of an event or experience.

## Participants

Purposive sampling combined with the maximum variation strategy and snowball sampling strategy was utilized to select participants from three tertiary hospitals and China Crohn’s & Colitis Foundation in Hangzhou, Zhejiang province. Purposive sampling was primarily used to select participants likely to provide valuable information, and maximum variation was ensured by considering participants’ sex, age, education level, disease duration, and treatment methods. At the same time, a snowball sampling strategy was employed, inviting interviewees to refer other individuals who met the inclusion and exclusion criteria, thereby expanding the participant pool. The inclusion criteria consisted of the following: (1) having a diagnosis of CD on the basis of the 2018 Chinese criteria (Wu et al., 2018), (2) being 18 years of age or older and willing to share one’s illness experiences, and (3) reporting a positive change following the diagnosis of CD (through a conversation with researchers). Patients with other life-threatening or mental illnesses were excluded. Upon completing the 15th interview, the researchers reported that the participants’ responses were repeated, with no new themes or subthemes emerging that were related to the manifestations of and factors influencing PTG (Saunders et al., 2018). The researchers interviewed four additional patients to confirm that the analysis had reached data saturation. Ultimately, a total of 19 patients were interviewed for the study.

A female postgraduate nursing student, CZ, who was involved in clinical practice at three tertiary hospitals and volunteering with the China Crohn’s & Colitis Foundation in Hangzhou, Zhejiang Province, approached eligible patients. The study was comprehensively explained to the patients, and they were orally invited to participate in face-to-face interviews. A total of 21 patients with CD were invited to participate in the interviews, and 2 declined due to physical discomfort.

## Data collection

Before the interviews, researcher CZ spent six months establishing a rapport with the recruited patients as an intern nurse and student volunteer by actively engaging in IBD patient education activities, including proactively providing disease-related information and answering questions about the disease. Under the guidance of YZ, CZ conducted in-depth interviews from December 2019 to October 2020. Before the formal interviews commenced, the purpose of the research was explained to the participants, along with the strategies that would be used to protect their privacy and anonymity. Following the acquisition of informed consent, the interviews began with casual conversation to help participants feel at ease, including questions such as, “Can you tell me the general history of your illness?”. The interview outline for this study was as follows: (1) How did you feel after being diagnosed with the disease? (2) What positive changes did you experience? (3) In your opinion, what factors influenced the aforementioned changes? The researcher encouraged the participants to express their views in their own words.

Face-to-face interviews (*n* = 6) took place in coffee shops or parks. Owing to the COVID-19 pandemic, video (*n* = 6) and telephone interviews (*n* = 7) were also conducted in quiet and private settings. Notably, telephone interviews are limited in their ability to acquire nonverbal and environmental information (Novick, 2008; Ward et al., 2015). To mitigate this limitation, we paid close attention to auditory cues during the interviews. Each participant was interviewed once in this study. The average duration of video and telephone interviews tended to be longer than that of other interview formats, but the levels of detail in the data were similar.

During the interviews, no one was present except the participant and interviewer. All interviews were audio-recorded and ranged in length from 45 to 105 minutes, with a median duration of 75 minutes. The researchers transcribed the interviews verbatim and recorded field notes within 24 hours of the interview to document the details of the observations, interactions, environment, and participants’ body language.

## Data analysis

After each interview, the audio recording was transcribed verbatim within 24 hours. Data analysis was performed simultaneously with data collection via the conventional content analysis method (Hsiu & Sarah, 2005). To ensure the reliability and accuracy of the coding results, the data analysis was carried out by three researchers. Chinese was used as the language for the analysis, and the codes and quotations were translated into English by the researcher.

All data analysis was performed under the guidance of YZ. First, three researchers (CZ, HL, DC) read the texts independently and immersed themselves in them. Second, two researchers (CZ, HL) manually performed the initial coding inductively and scanned the text line by line to identify preliminary coding units. Third, those two researchers (CZ, HL) combined coding units with common features to form themes and subthemes. Another researcher examined the formed codes, subthemes, and themes. The three researchers worked together to discuss and determine the coding and topics. Through this iterative process, a comprehensive summary was generated, and the final themes were formulated. Finally, the researchers defined the themes and subthemes, identified the corresponding examples from the text data, and selected representative quotations from the transcripts as examples to support the generated themes.

## Rigor

The rigour of this research was ensured through the following measures: (1) Prior to the formal interviews, two practice interviews were conducted, and the interview guide and data analysis methods were regularly reviewed and refined in consultation with an expert, and (2) the researchers maintained reflective journals to continually engage in self-reflection, which helped mitigate the influence of their personal experiences and any assumptions they might have had regarding the treatment experiences of the patients.

## Results

Nineteen patients with CD (11 men and 8 women) aged 19–50 years were recruited for this study. Eight participants (42%) were married. In terms of education, most participants held a bachelor’s degree (*n* = 12). With respect to occupation, there were 4 students, 10 employed individuals, 4 freelancers, and 1 unemployed participant. Only 4 participants identified with a religion, with 3 practicing Buddhism and 1 practicing Christianity. In terms of average monthly household income, the majority of patients, eight in total, earned more than 10,000 RMB. Ten participants had undergone surgery, with 8 having had one surgery, 1 having had three surgeries, and 1 having had four surgeries. According to disease activity indices, 15 participants were in remission at the time of the interview, whereas 4 were experiencing mild disease activity. The disease duration ranged from 8 months to 20 years. Table 1 presents the sociodemographic and clinical characteristics of the participants.

**Table 1.** Demographic and disease characteristics of the interview participants (*n* = 19).

NO.	Gender	Age	Marital status	Residence	Education level	Occupation	Religious beliefs	Per capita income (RMB)	Time of surgeries	Disease Activity Index	Course of disease (Months)
1	Female	25	Single	Zhejiang	Bachelor degree	Clerk	/	3000–5000	0	0	8
2	Male	20	Single	Zhejiang	Bachelor degree	Student	/	5001–10,000	1	6	36
3	Male	23	Single	Zhejiang	Bachelor degree	Student	/	3000–5000	1	0	129
4	Male	34	Single	Zhejiang	Bachelor degree	Clerk	/	5001–10,000	0	2	101
5	Female	45	Single	Guangdong	Associate degree	Freelance	Buddhism	3000–5000	0	1	240
6	Male	24	Single	Shanghai	Bachelor degree	Student	/	>10000	0	0	36
7	Male	27	Single	Shanghai	Master degree	Clerk	/	5001–10,000	1	5	82
8	Male	39	Married	Jiangsu	Bachelor degree	Engineer	/	>10000	0	1	18
9	Female	34	Married	Zhejiang	Bachelor degree	Freelance	Buddhism	3000–5000	1	0	84
10	Male	25	Single	Jiangsu	Bachelor degree	Unemployed	/	>10000	0	1	73
11	Male	32	Married	Jiangsu	Bachelor degree	Programmer	Christianity	>10000	0	0	120
12	Male	35	Married	Jiangsu	Associate degree	Engineer	/	>10000	0	0	74
13	Male	31	Single	Chongqing	Bachelor degree	Freelance	/	>10000	1	3	20
14	Female	44	Married	Zhejiang	Master degree	Teacher	Buddhism	5001–10,000	1	2	80
15	Female	19	Single	Anhui	Bachelor degree	Student	/	3000–5000	3	0	72
16	Male	47	Married	Zhejiang	Senior high school	Freelance	/	>10000	1	1	132
17	Female	39	Married	Zhejiang	Bachelor degree	Self-employed	/	>10000	0	0	84
18	Female	50	Married	Zhejiang	Master degree	Teacher	/	5001–10,000	1	5	144
19	Female	28	Single	Zhejiang	Associate degree	Clerk	/	3000–5000	4	6	109

The data analysis yielded two themes and eight subthemes. The two themes were as follows: (1) “Manifestations of PTG” and (2) “Factors influencing PTG”. The overall theme tree is shown in Table 2.

**Table 2.** Manifestations and factors influencing PTG among Chinese CD patients.

Theme	Sub-theme
Manifestations of PTG	Improving interpersonal relationships
	Enhancing personal strengths
	Changing life priorities
	Expanding possibilities
	Gaining religious faith
Factors influencing PTG	Individual factors
	Disease condition
	Social support

## Manifestations of PTG

The study revealed that patients’ PTG manifestations varied, mainly in the following five aspects: improving interpersonal relationships, enhancing personal strengths, changing life priorities, expanding possibilities and gaining religious faith (see Table 2).

## Improving interpersonal relationships

Improving interpersonal relationships is reflected in three main aspects: “valuing family”, “becoming more empathetic” and engaging in “altruistic behaviours”.

“Valuing family” refers to patients’ realization of the support their family has provided during their illness, the sincere efforts their family has made, and the importance of family relationships, resulting in their willingness to make changes to maintain and enhance their emotional ties with their family members.

Some patients (*n* = 10) reported that they had strengthened their contact with family members and placed greater emphasis on family relationships during the illness.

I feel that my family is very good to me, and kinship is very important; that is, family members should support each other… when I encounter something now, I will also be very considerate for my own family. (Interviewee 18)

“Becoming more empathetic” means that the patients began to think differently and empathize with others after being diagnosed with the disease rather than being solely self-centred.

Some patients (*n* = 11) felt that they were more understanding of others’ feelings in their interactions with others after being diagnosed with their illness.

I think it does make a difference in my interpersonal relationships. I mean that I used to care about myself a lot, but after this (the illness) … I try to think from other people’s perspectives.” (Interviewee 15)

Some patients (*n* = 12) developed “altruistic behaviours” because they had received help from others during their illness, and they wanted to give others more help in their daily lives.

The main idea is that you want to help others, because you have received help from others and you know the meaning of it, and you will have the idea that you should help others as much as you can. (Interviewee 5)

## Enhancing personal strengths

“Enhancing personal strengths” suggests that during their illness, patients experienced more difficulties, reported an improved mentality and ability to face setbacks, and experienced positive changes in their self-perception. This theme mainly includes three aspects: “improving self-efficacy,” “having a more peaceful mentality,” and “being more independent.”

“Improving self-efficacy” means that patients perceived that they had increased confidence in facing the difficulties brought about by the disease and dared to face problems head-on rather than choosing to avoid them as before.

The patients (*n* = 8) further improved their ability to cope with problems and were more courageous in facing and solving problems because they were continually forced to be proactive during their illness.

When I encounter something, I solve it, rather than running away. (Interviewee 10)

There were also some patients (*n* = 5) who felt that they had accumulated a great deal of experience by constantly solving problems and had become more proficient at dealing with various problems.

Because you have experienced something, you will know how to deal with it when your mentality is about to collapse or something like that next time. (Interviewee 4)

“Having a more peaceful mentality” means that the patients were able to view things more objectively and rationally and face their current situation with a more peaceful mentality.

The majority of patients (*n* = 15) felt that their mindset had changed considerably after the illness and that they were able to accept new things with a more tolerant mindset.

After surviving a serious illness, my whole personality seems to have changed. It’s just like you’ve gone through it, and you won’t freak out over trifling things anymore. (Interviewee 9)

“Being more independent” means that patients relied on their own strength to solve problems and became more autonomous.

In the past, I used to depend a lot on my boyfriend and would always want him to accompany me. But now, I can do things on my own. (Interviewee 1)

### Changing life priorities

“Changing life priorities” means that the patients reprioritize important things in their lives, mainly in the areas of “putting health first” and “doing what they want to do”.

Sometime after their diagnosis, some patients (*n* = 9) began to reflect on their past lifestyles and attitudes, and they realized the importance of health.

I realized that health is really important. While in the past, I may have thought that we should pursue many things and that younger people should enjoy themselves with feasting and other kinds of entertainment, chase after fame and wealth or something else. But now I have found that our health is the most important thing for us. (Interviewee 13)

The emphasis on health was also reflected in the fact that some patients prioritized their children’s health over school achievement, focusing more on their children’s physical and mental health rather than their course grades (*n* = 1).

After I became ill, I felt that grades weren’t the most important thing, and I wanted him to grow up happily, not just for the grades. (Interviewee 18)

Some patients (*n* = 6) followed their minds; they thought about what kind of life they truly wanted to live and did the things they truly wanted to do.

I feel that I have become more caring about myself. After being ill, I feel that I will listen more to my own heart and be moderate at times. I will eliminate things that aren’t important to me. (Interviewee 6)

## Expanding possibilities

“Expanding possibilities” refers to some patients who discovered a broader world and more possibilities and realized that they could do more meaningful things through hard work.

Some patients (*n* = 3) became volunteers in China Crohn’s and Colitis Foundation due to their illnesses; during this period, they had received volunteer training, which broadened their horizons and promoted their desire to become a better person.

I think that the foundation broadens my horizons because there are a lot of excellent people there, and it organizes many kinds of activities…I think it can make me a better person. (Interviewee 19)

Some patients (*n* = 3) became more aware of areas they had previously not paid attention to due to their illness and discovered that they could expand their existing work and personal lives to encompass a larger scope, giving them new goals and possibilities in life.

I only focused on things like taking care of my students during class and taking care of my family before. Now, I realize that the world outside is actually very big and there are many people. Our group is very large and there are many things I can do. (Interviewee 14)

## Gaining religious faith

“Gaining religious faith” means that the patients had experiences related to existence, spirituality and religion after their diagnosis with the illness and gained a new understanding of the illness, the meaning of life, and life in general.

Some patients (*n* = 2) gained religious faith after their illness, which helped them find purpose and meaning in life and a spiritual resting place.

I am a Christian who goes to prayer and reads the Bible every day. I think that when I really establish a belief, and then finding meaning in life, finding my direction, my whole person changes. (Interviewee 14)

One patient reported that he had a different understanding of disease and life after being diagnosed with the illness and was more motivated to live a good life, relying on faith.

I happened to come across Buddhism. If I didn’t have this faith, then I die when I am dead……but now that I have this faith, I have time to do what I can do. It becomes a strong support behind me. (Interviewee 14)

## Factors influencing PTG

The factors that contribute to growth after trauma in CD patients mainly include individual factors, social support, and disease condition, which can play different roles in a patient’s growth at various stages (see Table II).

## Individual factors

The influence of individual factors on CD patients’ growth mainly refers to patients’ personality traits, self-reflection, and coping strategies.

“Personality traits” refer to people’s long-term formation of relatively stable personality characteristics and behaviour patterns.

Some patients expressed that their optimistic attitude and positive mindset towards their illness enabled them to accept their illness quickly and grow from it.

It seemed like I have a positive attitude, and thus I accept the disease very quickly. (Interviewee 1)

Other patients displayed resilience when facing their illness, bravely confronting the challenges it brought, which ultimately led to growth.

In that moment, the illness might have knocked me down, but as long as I have a breath left, I rely on my own inner strength and external help from doctors and fellow patients. As long as I can stand up, I will be stronger than before, and my tolerance will also increase. (Interviewee 14)

“Self-reflection” refers to CD patients examining their original ideas and behaviours under the guidance of those ideas and then changing their own cognition and adjusting their behaviours.

Some patients adjusted their cognition through self-reflection to reinterpret the disease and found benefits from this activity.

Being sick is not necessarily a bad thing; it may be a reminder that your body is already being pushed to its limits and that it’s time to start taking care of yourself. (Interviewee 12)

Some patients reflected on their previous lives, rebuilt their understanding of life and grew from it.

It was when I was hospitalized that I stopped and I realized how busy I had been in recent years…. I then felt whether I really had been too busy before and hadn’t left a little bit of time. I allowed myself to meditate and realize what kind of life I really wanted to live by myself. (Interviewee 14)

“Coping strategies” refer to the patient’s perspective on maintaining psychological equilibrium and problem solving during the disease process; this theme includes two subthemes: “emotional coping” and “problem-solving coping”.

“Emotional coping” refers to the methods that some patients use to regulate their emotions, such as focusing on something other than the illness or venting negative emotions, to help them relieve their pain and carry out constructive thinking.

Don’t dwell on the fact that you’re sick and feel like the world has abandoned you, do something else and you’ll forget about it; then you’ll be calmer, and your thoughts will become clearer, making it easier to face the disease.” (Interviewee 1)

There were also patients who were confronted with stressful situations and strove to solve problems by reassessing their surroundings and actively searching for external or internal resources that were conducive to problem solving to achieve growth.

Don’t overthink it; it’s that simple. When you encounter a problem, go solve it. You’ll gain experience in the process, and you’ll progress from where you used to be. (Interviewee 14)

### Disease condition

The alleviation and exacerbation of the disease are important factors that affect CD patient growth after trauma. Improvements in or worsening of a patient’s condition could promote growth in certain situations.

Some patients mentioned that their mental state changed once their condition improved. Once their condition improved, they were able to view their illness more rationally, reflect on the past, and gain personal growth.

It depends on your body recovered and your brain starts functioning properly again, allowing you to focus on and reflect on past events. (Interviewee 5)

Some patients expressed that although the initiation of the disease caused them pain, it also made them more eager to acquire relevant knowledge and face the disease actively.

The most critical year for me was after the relapse in 2019 and the three operations that followed. It made me even more eager to understand the nature and temperament of CD, it made me more positive. (Interviewee 15)

As the disease progressed, the patients became more accepting of it by proactively or passively seeking more knowledge and experiences related to the disease. They gradually learned how to cope better with the disease.

Because I have been sick for a long time, I have paid more attention to this area and have more knowledge than before. My mindset has gradually changed since then. (Interviewee 12)

#### Social support

Social support refers to the external support that patients receive during their illness, including “family support,” “peer support,” and “medical support”.

“Family support” refers to the care, financial support, and emotional support that family members can provide when a patient seeks medical treatment.

My parents said no matter how expensive the treatment would be, even if they had to sell their house, they would still take me to see a doctor. It touched me deeply. They are a great support behind me, which empowers me to face the disease better. (Interviewee 9)

“Peer support” refers to the emotional and informational support provided by friends and wardmates. CD patients often reported that communication between wardmates conveyed practical and effective information and experiences with medical care and that the understanding and support of friends could help patients resolve their immediate difficulties while also helping them gain emotional strength and motivation for their growth.

I often feel very close to some wardmates. They are like my families. We chat together without any distance, and we can understand each other well. (Interviewee 19)

“Medical support” refers to the medical and emotional support provided by healthcare professionals to CD patients.

Doctors and professors, they are also particularly concerned about me, and so as the nurses, they help me change the medication carefully, and then there are times when they also said that you can listen to music or you can hum a song, to distract your attraction, and then my mentality becomes more positive. (Interviewee 15)

## Discussion

### PTG in CD patients

This study identified five aspects of PTG in CD patients: improving interpersonal relationships, enhancing personal strengths, changing life priorities, expanding possibilities, and gaining religious faith. These findings are generally consistent with previous research (Ibrahim et al., 2022; Pakenham, 2007; Purc-Stephenson et al., 2015; Zhai et al., 2019). However, we found that the characteristics of CD as a disease and Chinese cultural factors have multiple influences on PTG in CD patients.

Previous studies have shown that survivors of acute trauma (Nishi et al., 2010) or cancer (Morris et al., 2012) often appreciate positive aspects of life. However, similar phenomena are less common among CD patients. This is because survivors of acute trauma or cancer undergo more profound introspection and appreciation of positive aspects of life while directly facing life-threatening situations. This awareness triggers a heightened sensitivity to and appreciation for the positive aspects of life. In contrast, CD patients, owing to long-term life restrictions and the irreversible nature of their disease, often find it difficult to experience the same mentality as survivors do. They tend to perceive themselves primarily as CD patients living with their illness. This difference in mindset may present greater challenges for CD patients in experiencing and appreciating positive aspects of life.

Compared with patients with other chronic illnesses in China (Wang et al., 2011), CD patients contemplate the philosophical aspects of life less. Only a small number of patients explored and interpreted the meaning of life through religious beliefs. This may be related to the severity of the disease and the influence of religion. In a study of Chinese patients with spinal cord injury, patients reflected on the meaning of their existence (Y. Wang et al., 2017). This may be related to the fact that spinal cord injury poses a greater threat than CD does, with patients having a closer proximity to death. The resulting disability caused by spinal cord injury presents greater obstacles for patients to reintegrate into society, impacting their ability to realize their self-worth and leading to a greater inclination to question and explore the meaning of life. Furthermore, researchers such as Ibrahim (Ibrahim et al., 2022) studied individuals in Egypt who experienced psychological health issues and reported that most participants emphasized the importance of spirituality and religious beliefs. This is related predominantly to the Muslim population in Egypt, where relying on religious resources to cope with adversity is common. When the Chinese version of the PTG Inventory was revised, Chinese researchers, such as Wang (Y. Wang et al., 2017), reported that the Chinese understanding of the concept of “faith” differs from that of many religious groups in other countries. This suggests significant differences in the Chinese cultural attitudes towards religion and faith compared with those in other countries. These cultural factors suggest that gaining faith or belief is only important for the minority of patients who reported it as a significant factor.

Prosocial behaviours often emerge among survivors of disasters such as earthquakes and accidents, a phenomenon referred to as “altruism born of suffering” in the academic community (Staub & Vollhardt, 2008). Similarly, clear observations of altruistic behaviour were observed among Chinese CD patients. In comparison, a Canadian study on PTG in IBD patients reported an increase in empathy levels but did not find corresponding altruistic behaviours (Purc-Stephenson et al., 2015). This difference may be related to the characteristics of Chinese culture, where individual behaviour is closely linked to the welfare of the social community (Jackson & Wang, 2013). Chinese culture emphasizes social collective consciousness, and individual behaviour is considered part of social responsibility. Individuals are encouraged to prioritize the collective interests of society and exhibit altruistic behaviour. Such behaviour not only improves the individual’s own condition but also contributes to the collective well-being of CD patients, allowing them to form a stronger social support network and providing them with more support and resources.

With respect to the expansion of possibilities, these CD patients developed new interests or pursued opportunities for further education and new careers, which is similar to findings in previous research (Muller et al., 2010). Compared with type 1 diabetes patients (Feng & Zou, 2021), CD patients are more willing to explore new possibilities in life and exhibit a greater level of motivation for their future. In contrast, diabetes patients prefer to continue living in their existing state or make further plans for their lives without expressing a strong desire for new things or skills (Feng & Zou, 2021). This may be related to CD patients having closer connections with fellow patients and volunteers and their environment and external information being more positive. This finding suggests the need to establish diverse peer support platforms for CD patients in the rehabilitation process to provide a rich and inclusive social environment. This also tells us that we should emphasize communication and experience sharing among patients and peers, which is important to help patients to face challenges and experience growth through the power of role models and to inspire patients to explore their own potential.

## Factors influencing PTG in CD patients

The results of this study reveal the influence of individual factors, social support, and the disease condition on PTG in CD patients. Although previous quantitative studies have explored the complex pathways underlying PTG in IBD patients, including the relationships among disease cognition, PTG, and quality of life (Hamama Raz et al., 2021), this study provides a more comprehensive understanding of the influencing factors based on interviews.

Individual factors such as optimistic and resilient personality traits facilitate growth in CD patients. This finding is consistent with the mention of pretrauma extraversion as a factor related to PTG in Calhoun and Tedeschi’s model of PTG (Tedeschi et al., 2018). Similar findings have been reported in other chronic disease populations, such as patients with breast cancer and multiple sclerosis (Büyükaşik-Colak et al., 2012; Zeltser, 2017), for whom optimistic and resilient personality traits promote PTG. This may be because personality traits can influence the symptom experience and perception of chronic disease patients, and patients with optimistic personality traits can adjust their negative emotions in a timely manner and actively accept and face their illness (Anzaldi & Shifren, 2019). The influence on PTG in CD patients of other personality factors related to the Big Five personality traits requires further investigation. Positive coping styles are important for the progression of CD patients towards growth. This factor has also been validated in previous quantitative research on the benefits for IBD patients (Muñoz González et al., 2023). CD patients often employ positive coping strategies such as emotion-focused coping and problem-focused coping. However, in Chinese society, emotion-focused coping can be influenced by “face” culture (Shi-xu, & Feng-Bing, 2013). Expressing negative emotions openly in front of nonfamily members or friends is often seen as losing face, which may damage harmonious interpersonal relationships (Kinnison, 2017). Self-reflection is a deep cognitive processing method that propels patients towards self-growth through purposeful rumination (Wittmann et al., 2009). It also plays an important role in the process of modifying core beliefs in patients with other chronic diseases (Iaquinta & Larrabee, 2004; Wittmann et al., 2009). Therefore, we suggest that future positive psychological interventions incorporate self-expression techniques such as expressive writing and narrative nursing to facilitate emotional expression, promote self-reflection among patients, and ultimately help Chinese chronic disease patients grow.

Social support from family, peers, and healthcare professionals provides CD patients with material, emotional, and informational resources that influence their coping strategies. Multiple studies in positive psychology support this perspective in various chronic disease populations (Esposito, 2016; Pakenham, 2007; Sato et al., 2008). In Eastern countries, which are influenced by collectivist cultures, individuals place greater emphasis on social support and interaction (Triandis, 2018). In China, “illness is a family affair”(Jackson & Wang, 2013). Family support can alleviate individuals’ psychological burdens, promote the recovery of patients’ physical and psychological health, and facilitate their disease management. Currently, peer support among CD patients relies heavily on mutual communication and information sharing among fellow patients, with limited support from the general public. This situation is partly attributed to the relatively low awareness of IBD among the Chinese public and the limited experience with CD patients (Xu et al., 2022). Therefore, efforts are needed to strengthen disease awareness regarding IBD. Additionally, professional support from healthcare institutions is crucial for providing substantial guidance to CD patients (Li et al., 2020). In the future, introducing telemedicine and online consultation platforms will help patients manage their diseases better.

The results of this study indicate that both the alleviation and exacerbation of patients’ conditions can contribute to their personal growth. On the one hand, the relief of symptoms reduces patients’ suffering, allowing them to shift their focus from passively dwelling on the disease and its associated pain to actively contemplating the challenges posed by the illness. Similar findings have been reported in other common chronic disease populations (Purc-Stephenson, 2014). On the other hand, the worsening of symptoms prompts patients to seek a deeper understanding of their condition, facilitating personal growth through the process of learning. Similar conclusions were reached by the Canadian scholar Purc-Stephenson (Purc-Stephenson, 2014), who reported that IBD patients had higher levels of PTG than those with arthritis. This may be attributed to the greater destructiveness or greater emotional impact of chronic diseases, which could be associated with higher levels of PTG. Similar effects can also be observed with the progression of the disease. Over time, patients gradually acquire knowledge and experience related to the illness, either actively or passively, and learn how to cope with the disease, leading to personal growth. A longitudinal study conducted in the United Kingdom (Sirois & Hirsch, 2017) revealed a significant correlation between the progression of IBD and increased scores in two dimensions of psychological thriving: life satisfaction and self-improvement. Patients who learn more about their illness due to worsening symptoms or disease progression may also reduce their sense of helplessness in the face of the disease. Research has shown that reducing feelings of helplessness contributes to PTG in IBD patients (Hamama-Raz et al., 2021). Future studies could explore the multiple forms of relationships among disease severity, disease progression, feelings of helplessness, and PTG in patients with CD.

## Limitations

There are several limitations of this study. First, these participants may not represent the general CD population, as they were all in remission or experiencing mild disease and had relatively high education levels. Second, video and telephone interviews were adopted in some cases because of the impact of the COVID-19 pandemic, which may have affected the acquisition of nonverbal and environmental information during the interviews.

## Conclusion

To our knowledge, this study represents the first qualitative study on PTG in Chinese patients with CD and the factors influencing it. The manifestations of PTG in Chinese CD patients include improving interpersonal relationships, enhancing personal strength, changing life priorities, expanding possibilities and gaining religious faith. The influencing factors include individual factors, disease conditions, and social support. Additionally, this study focused on the multiple influences of Chinese cultural characteristics on CD patients, providing a comprehensive understanding of the unique experiences of this population in terms of PTG. These findings provide important insights for clinical research on PTG in Chinese CD patients and the development of comprehensive intervention strategies.

## Data Availability

The data that support the findings of this study are available on request from the corresponding author or first authors on reasonable request. The data are not publicly available due to ethical issues.
